# Investigating factors associated with success of breastfeeding in first-time mothers undergoing epidural analgesia: a prospective cohort study

**DOI:** 10.1186/s13006-018-0184-7

**Published:** 2018-09-05

**Authors:** Daryl Jian An Tan, John Paul Lew, Maria Binte Jumhasan, Cynthia Pang, Rehena Sultana, Ban Leong Sng

**Affiliations:** 1 Duke-NUS Medical School, Singapore, Singapore; 2 Department of Anaesthesiology, Singapore General Hospital, Singapore, Singapore; 3 Division of Nursing, KK Women’s and Children’s Hospital, Singapore, Singapore; 4 Centre for Quantitative Medicine, Duke-NUS Medical School, Singapore, Singapore; 5 Department of Women’s Anaesthesia, KK Women’s and Children’s Hospital, Singapore, Singapore

**Keywords:** Breastfeeding, Risk factors, Predictors, Cohort study, Singapore

## Abstract

**Background:**

We investigated the possible risk factors that could influence the likelihood of breastfeeding at 5 to 9 weeks postpartum with our primary aim being to analyse the associations between psychological vulnerabilities, such as peripartum depression and anxiety, and continued breastfeeding. Our secondary aim was to investigate other non-psychological factors’ influence on continued breastfeeding.

**Methods:**

A prospective cohort study was conducted in KK Women’s and Children’s Hospital in Singapore. Healthy nulliparous parturients at ≥36 weeks gestation with a singleton fetus who received epidural analgesia were recruited. Demographic and anaesthetic data were obtained. Self-reported psychological and pain determinants such as anxiety (State-Trait Anxiety Inventory), depression (Edinburgh Postnatal Depression Scale), stress (Perceived Stress Scale), pain susceptibility (Pain Catastrophizing Scale) and pain perception (McGill Pain Questionnaire) were also recorded at baseline. A phone interview was then performed at 5 to 9 weeks postpartum to obtain information on breastfeeding status.

**Results:**

329 participants were included into this study, of which 263 (79.9%) of them were still breastfeeding at 5 weeks postpartum. Multivariate logistic regression analysis showed that a higher State-Trait Anxiety Inventory score (Adjusted Odds Ratio [AOR] 0.97; 95% Confidence Interval [CI] 0.94, 1.00) at baseline, higher intrapartum blood loss (AOR 0.76; 95% CI 0.61, 0.93), and occurrence of fetal anomalies (AOR 0.15; 95% CI 0.03, 0.72) were associated with reduced likelihood of breastfeeding at 5 to 9 weeks postpartum. Indians (AOR 0.56; 95% CI 0.20, 1.53), Malays (AOR 0.30; 95% CI 0.14, 0.62) and other ethnicities (AOR 0.36; 95% CI 0.16, 0.83) were less likely to continue breastfeeding compared to Chinese participants. On the other hand, receiving any support services on breastfeeding during the participants’ hospital stay was 3.3 times more likely (AOR 3.30; 95% CI 1.21, 9.02) to increase the likelihood of breastfeeding at 5 to 9 weeks postpartum.

**Conclusion:**

We identified 5 independent association factors that could have significant influences on breastfeeding at 5 to 9 weeks postpartum. Healthcare providers could utilize this risk stratification to identify parturients likely to have poorer breastfeeding outcomes and undertake interventions that may help safeguard optimization of breastfeeding outcomes and parturient care.

**Trial registration:**

Clinicaltrials.gov NCT02278601. Registered 26 October 2014.

**Electronic supplementary material:**

The online version of this article (10.1186/s13006-018-0184-7) contains supplementary material, which is available to authorized users.

## Background

The 2011 National Breastfeeding Survey for Singapore, which included 1962 new mothers from nine hospitals in Singapore, found that while 96% of the new mothers were breastfeeding on the day of discharge, only 80% and 42% of them were still breastfeeding at 2 months and 6 months respectively [1]. This is despite the fact that most Singaporean mothers know that “breastfeeding is the best form of feeding for a newborn” (93%), “breast milk is the best for baby” (79%) and that “breast milk protects baby from a wide range of disease” (54%) [1]. The most commonly cited reasons for stopping breastfeeding included being “not able to supply enough milk” (61%) and the “need to return to work” (24%) [1]. Postpartum anxiety and depression have been shown to have negative associations to continued breastfeeding outcomes [2–4]. Women with symptoms of postpartum anxiety are less likely to initiate breastfeeding and, if breastfeeding were to be commenced, are more likely to terminate breastfeeding earlier and supplement with infant formula [2]. Postpartum depression is also negatively associated with decreased breastfeeding duration, increased breastfeeding difficulties and decreased levels of breastfeeding self-efficacy [3, 4].

While there have been various studies that demonstrate associations between postpartum anxiety and depression and the likelihood of breastfeeding, the relationship between peripartum anxiety and depression and its effects on breastfeeding has not been well researched. We hypothesize that the psychological vulnerabilities the mother experiences during delivery, particularly peripartum anxiety and depression, can have negative impacts on the mother’s inclination to breastfeed. The primary aim of this study is to investigate the associations between psychological vulnerabilities, specifically peripartum anxiety and depression, and the likelihood of breastfeeding at 5 to 9 weeks postpartum. The secondary aim of this study is to investigate demographic, obstetric and pain characteristics that may also influence breastfeeding at 5 to 9 weeks postpartum.

## Methods

KK Women’s and Children’s Hospital (KKH) is a tertiary level care hospital in Singapore that provides specialized medical care to the obstetric, gynaecological and pediatric populations. This cohort study uses data collected in an ongoing trial: The Collaborative Outcomes with Labour Epidural Use Study (COLEUS). The COLEUS study is registered on clinicaltrials.gov (NCT02278601). The main study is a double blinded, randomized controlled trial, with the primary aim being to investigate the efficacy of 3 different epidural maintenance regimens.

The study population included healthy (American Society of Anesthesiologists physical statuses 1 and 2), nulliparous, adult parturients at ≥36 weeks gestation, carrying a singleton fetus, presenting in early labour (cervical dilation ≤5 cm), requesting labour epidural analgesia and who were suitable for a combined spinal-epidural technique. Parturients with multiple pregnancies, a non-cephalic fetal presentation, a history of obstetric or medical complications, contraindications to neuraxial blockade, or who had received parenteral opioids within the last 2 h or had an unintentional dural puncture at the initiation of the epidural were excluded. The participants, attending midwives and medical team were all blinded to the participant assignment and treatment allocation.

Self-reported questionnaires on data of anxiety (State-Trait Anxiety Inventory (STAI)), depression (Edinburgh Postnatal Depression Scale (EPDS)), stress (Perceived Stress Scale (PSS)), pain susceptibility (Pain Catastrophizing Scale (PCS)) and pain perception (Short Form McGill Pain Questionnaire (SFMPQ)) were collected from the participant at baseline after they had just received labour epidural analgesia. We selected to use these questionnaires because they were not only convenient for the participants, but also the reliability and validity of the questionnaires used in our study have been well-established [5–10]. Participant demographics, obstetric and anaesthetic data were also collected in this study.

A phone interview would be done at 5 to 9 weeks postpartum. This time period was selected to assess the short term breastfeeding status of participants, as well as to screen for postnatal depression. The participant would be asked to complete another series of questionnaires including the EPDS, STAI, and the Breastfeeding Questionnaire via a phone interview conducted by a blinded research study team member. The Breastfeeding Questionnaire has been included in this manuscript (see Additional file 1). All data collected was entered into an electronic REDCap database, by two research team members and subsequently crosschecked after entry, to ensure the accuracy of the data recording. Any discrepancies and inconsistencies in the data entry were reviewed and de-conflicted by a third investigator. Participants who responded to this 5 to 9 weeks postpartum phone interview were included into the analysis.

### Sample size calculation and statistical analysis

The planned sample size for the study was 320 participants, which is based on the following assumption: 80% of mothers will continue breastfeeding till 2–4 months after delivery based on the National Breastfeeding Survey conducted in Singapore [1], with a 95% confidence interval (CI) (precision) of 79.96% to 80.04% i.e. a width of confidence interval of 0.086%, using the Wilson score interval method for CI calculation [11, 12]. Our primary objective was to investigate for possible determinants that may influence the mother’s likelihood of breastfeeding at 5 to 9 weeks postpartum. Peduzzi et al., Concato et al. and Vittinghoff et al. recommended that multivariable logistic regression models should be used with at least 10 events per predictor variable [13–15]. From our data, the prevalence of participants who breastfed at 5 to 9 weeks postpartum was 82.4% (263/319). Based on the recommendations, we could adjust for a maximum of 263/10 ≈ 26 variables in the multivariate model. Our study was adequately powered (> 80%) with 320 patients based on following assumptions: proportion of participants who “breastfed 5 to 9 weeks postpartum” as 80%, odds ratio (OR) of 3.5 (or 0.29) and alpha or type I error rate as 5%.

The primary outcome “*status of continued breastfeeding*” was treated as binary data with categories of “*yes*” or “*no*”. Demographic, clinical, self-reported questionnaires, obstetric and anesthetic data were summarized as mean with standard deviation (SD) for continuous variables, and frequency with corresponding proportion for categorical variables. Univariate and multivariate logistic regression models were used to identify possible risk factors of breastfeeding at 5 to 9 weeks postpartum. Associations drawn from the logistic regression models were characterized using ORs with corresponding 95% CI. Variables with *p*-values < 0.20 in the univariate analysis and clinically important variables were selected for the multivariate logistic regression model. The union of the variables from forward, backward and stepwise method were used to finalize the list of variables in the multivariate model with entry and stay criteria as 0.2 and 0.05 respectively. Then we used likelihood ratio test followed by area under the curve (AUC) to determine the final multivariate model. The variables identified in the multivariate analysis were further analysed for the strength of their associations to the likelihood of breastfeeding at 5 to 9 weeks postpartum through a Receiver Operating Characteristic (ROC) curve. AUC from ROC was also reported. The significance level was set at 0.05 and all tests were two-tailed. Data were analysed using SAS version 9.3 software (SAS Institute Inc.; Cary, NC, USA).

## Results

**Fig. 1:**
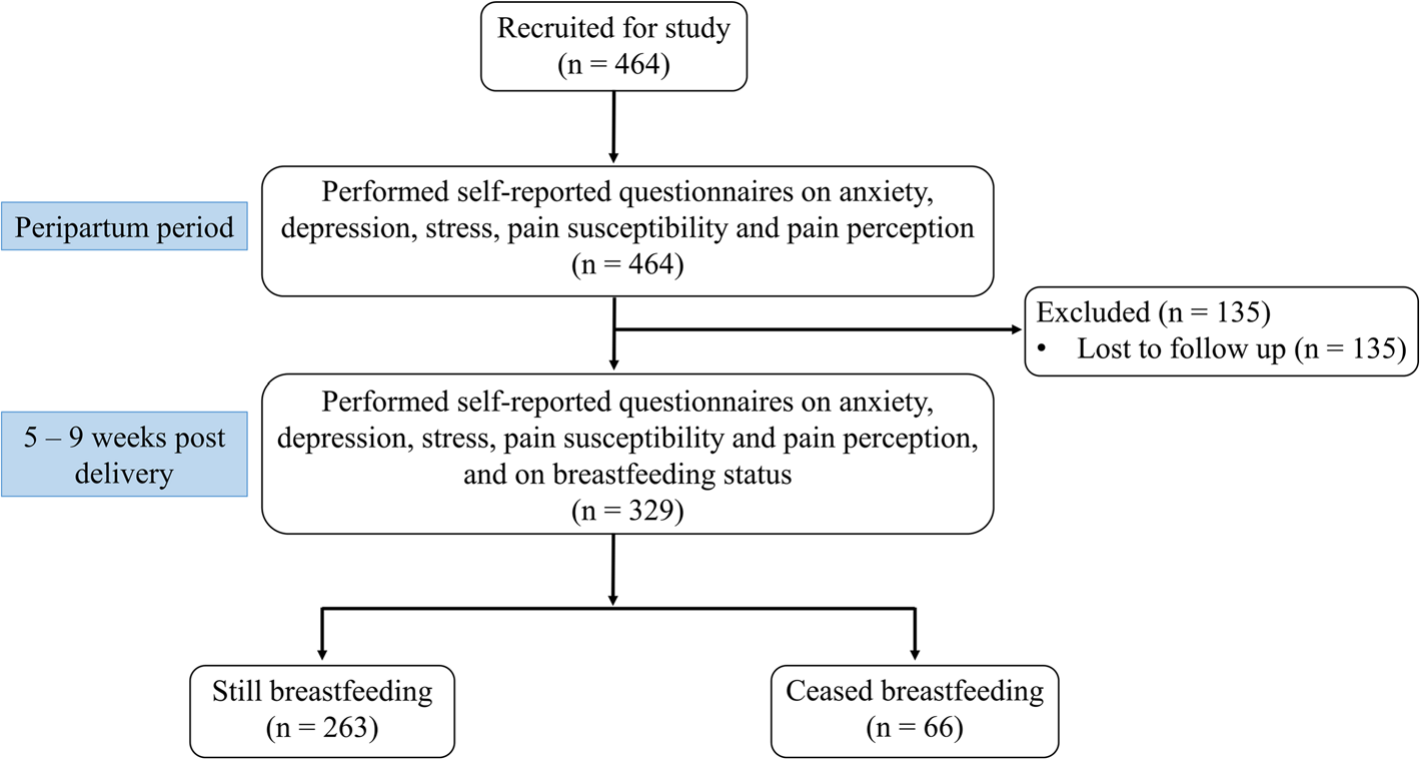
Flow chart of study

A total of 464 participants were selected for this study, of which 135 participants were lost to follow up and hence were not included into the study. The participant demographic characteristics are shown in Table 1. We found that 263 (79.9%) of the participants were still breastfeeding at 5 weeks postpartum. The proportions of those who breastfed at 5–9 weeks postpartum in each ethnic group were 84.6% (176/208) Chinese, 80.0% (24/30) Indian, 66.0% (33/50) Malay and 73.2% (30/41) of other ethnicities. Figure 1 illustrates the flowchart of the study.

**Table 1:** Participants’ demographic and obstetric characteristics (*n* = 329)

Characteristics	Participant still breastfeeding 5–9 weeks postpartum?		Total (*n* = 329)
	Yes (*n* = 263)	No (*n* = 66)	
Age (years)	30.2 ± 4.15	29.5 ± 5.58	30.1 ± 4.47
Ethnicity			
Chinese	176 (66.9)	32 (48.5)	208 (63.2)
Indian	24 (9.1)	6 (9.1)	30 (9.1)
Malay	33 (12.5)	17 (25.8)	50 (15.2)
Others	30 (11.4)	11 (16.7)	41 (12.5)
Mode of delivery			
Normal vaginal delivery	153 (58.2)	34 (51.5)	187 (56.8)
Instrument-assisted delivery	46 (17.5)	9 (13.6)	55 (16.7)
Emergency Caesarean Section	64 (24.3)	23 (34.8)	87 (26.4)
Satisfaction with birth experience			
Extremely satisfied	42 (16.0)	11 (16.7)	53 (16.1)
Very satisfied	74 (28.1)	17 (25.8)	91 (27.7)
Satisfied	126 (47.9)	32 (48.5)	158 (48.0)
Unsatisfied	19 (7.2)	3 (4.5)	22 (6.7)
Extremely unsatisfied	2 (0.8)	3 (4.5)	5 (1.5)
Complications experienced intrapartum			
Premature rupture of membranes	39 (14.8)	15 (22.7)	54 (16.4)
Maternal pyrexia	72 (27.4)	21 (31.8)	93 (28.3)
Non-reassuring fetal status	27 (10.3)	5 (7.6)	32 (9.7)
Failure to progress	32 (12.2)	13 (19.7)	45 (13.7)
Intrapartum blood loss (per 100 ml)	2.5 ± 1.04	3.0 ± 1.76	2.6 ± 1.23
Occurrence of fetal anomalies	3 (1.1)	4 (6.1)	7 (2.1)

Values are represented either as mean ± SD or number (proportion)

We also found that 7 (2.1%) participants had newborns with congenital disorders, of which 4 newborns had cardiac anomalies, 2 newborns had orthopedic anomalies and 1 newborn had neurological anomalies. When grading their birth experience, 91.8% of participants were above satisfied.

Results from self-reported questionnaires are shown in Table 2. From the Breastfeeding Questionnaire, 94.2% of participants had some form of breastfeeding support, of which help from the nurses in the maternity ward (82.1%) and from a lactation consultant (37.4%) were the most common. The mean infant age for cessation of breastfeeding was 34 days. The most common reasons for cessation reported by participants were having insufficient breast milk (75.8%) and choosing to stop breastfeeding (33.3%).

**Table 2:** Results from self-reported questionnaires collected from participants at baseline after they had just received labour epidural analgesia and at 5–9 weeks postpartum

Characteristics	Participant still breastfeeding 5–9 weeks postpartum?		Total (*n* = 329)
	Yes (*n* = 263)	No (*n* = 66)	
State-Trait Anxiety Inventory			
At baseline			
State anxiety score	36.0 ± 8.93	38.2 ± 12.55	36.4 ± 9.77
Trait anxiety score	37.5 ± 6.41	38.8 ± 8.56	37.7 ± 6.90
Total score	73.5 ± 14.19	77.0 ± 19.87	74.2 ± 15.51
At 5–9 weeks postpartum			
State anxiety score	30.0 ± 8.88	30.7 ± 9.48	30.2 ± 8.99
Trait anxiety score	35.0 ± 7.14	36.0 ± 8.00	35.2 ± 7.32
Total score	65.1 ± 15.21	66.7 ± 16.72	65.4 ± 15.51
Edinburgh Postnatal Depression Scale			
At baseline	7.0 ± 3.76	7.7 ± 5.33	7.2 ± 4.13
At 5–9 weeks postpartum	2.7 ± 3.79	3.3 ± 4.35	2.8 ± 3.91
Short-form McGill Pain Questionnaire (at baseline)	42.4 ± 34.30	48.5 ± 41.74	43.7 ± 35.99
Perceived Stress Scale (at baseline)	18.9 ± 4.60	19.3 ± 5.21	19.0 ± 4.72
Pain Catastrophizing Scale (at baseline)			
Rumination score	8.2 ± 4.34	8.7 ± 4.67	8.3 ± 4.40
Magnification score	4.3 ± 2.86	5.1 ± 3.15	4.4 ± 2.93
Helplessness score	9.4 ± 6.17	10.1 ± 6.06	9.6 ± 6.15
Total score	22.6 ± 12.59	24.5 ± 12.67	23.0 ± 12.61
Use of support services on breastfeeding (at 5–9 weeks postpartum)			
Help from lactation consultant during hospital stay	105 (39.9)	18 (27.3)	123 (37.4)
Help from ward nurse during hospital stay	218 (82.9)	52 (78.8)	270 (82.1)
Attended lactation clinic	12 (4.6)	1 (1.5)	13 (4.0)
Contacted hospital helpline	5 (1.9)	2 (3.0)	7 (2.1)
Not at all	11 (4.2)	8 (12.1)	19 (5.8)
Reported reasons for stopping breastfeeding (at 5–9 weeks postpartum)			
Insufficient milk supply		50 (75.8)	
Participant’s volition		22 (33.3)	
Difficulty with latching onto breast		8 (12.1)	
Medical reasons (either baby or mother)		6 (9.1)	
Fatigue/Tiredness		5 (7.6)	
Need to return to work after maternity leave		6 (9.1)	
Sore nipples		1 (1.5)	
Lack of breastfeeding facilities at workplace		1 (1.5)	

Values are represented either as mean ± SD or frequency (proportion)

The univariate and multivariate regression analyses are shown in Table 3. Covariates that are independently associated with breastfeeding at 5 to 9 weeks postpartum are: receiving any support services on breastfeeding (*p* = 0.02), achieving a lower state anxiety score at baseline (*p* = 0.03), not having any form of fetal anomalies (*p* = 0.02) and achieving lesser intrapartum blood loss (*p* = 0.01). Ethnic differences are also independently associated with the likelihood to breastfeed at 5 to 9 weeks postpartum (*p* = 0.01). Indians (Adjusted OR [AOR] 0.56; 95% CI 0.20, 1.53), Malays (AOR 0.30; 95% CI 0.14, 0.62) and other ethnicities (AOR 0.36; 95% CI 0.16, 0.83) were less likely to breastfeed at 5 to 9 weeks postpartum as compared to Chinese participants. The AUC based on multivariate analysis was 0.7065, demonstrating that the association factors utilised in this study and the identified covariates are moderately associated with breastfeeding at 5 to 9 weeks postpartum (see Additional file 2).

**Table 3:** Univariate and multivariate logistic regression analysis of covariates of breastfeeding 5 to 9 weeks postpartum

Characteristics	Unadjusted OR (95% CI)	*P* – value	Adjusted OR (95% CI)	*P* – value
Age (years)	1.036 (0.975, 1.102)	0.2545		
Mode of delivery (Ref: Normal vaginal delivery)		0.2180+		
Instrument-assisted delivery	0.618 (0.338, 1.132)	0.0872		
Emergency Caesarean Section	1.136 (0.508, 2.541)	0.3528		
Race (Ref: Chinese)		0.0217+		0.0052+
Indian	0.727 (0.276, 1.920)		0.557 (0.203, 1.528)	0.7416
Malay	0.353 (0.176, 0.708)		0.294 (0.140, 0.618)	0.0668
Others	0.496 (0.226, 1.089)		0.362 (0.157, 0.833)	0.3129
State-Trait Anxiety Inventory				
At baseline				
State anxiety score	0.977 (0.951, 1.004)	0.1002	0.968 (0.940, 0.997)	0.0304
Trait anxiety score	0.973 (0.935, 1.011)	0.1628		
Total score	0.985 (0.969, 1.003)	0.0977		
At 5–9 weeks postpartum				
State anxiety score	0.993 (0.964, 1.022)	0.6244		
Trait anxiety score	0.982 (0.948, 1.018)	0.3180		
Total score	0.994 (0.977, 1.010)	0.4501		
Edinburgh Postnatal Depression Scale				
At baseline	0.961 (0.902, 1.025)	0.2306		
At 5–9 weeks postpartum	0.966 (0.905, 1.031)	0.3002		
Short-form McGill Pain Questionnaire	0.996 (0.988, 1.003)	0.2472		
Perceived Stress Scale	0.985 (0.929, 1.044)	0.6133		
Pain Catastrophizing Scale				
Rumination score	0.977 (0.919, 1.039)	0.4576		
Magnification score	0.907 (0.830, 0.992)	0.0332		
Helplessness score	0.982 (0.940, 1.025)	0.4010		
Total score	0.989 (0.968, 1.010)	0.2896		
Complications experienced intrapartum (Ref: No)				
Premature rupture of membranes	0.592 (0.303, 1.155)	0.1243		
Maternal pyrexia	0.808 (0.450, 1.449)	0.4742		
Non-reassuring fetal status	1.395 (0.516, 3.774)	0.5115		
Failure to progress	0.565 (0.278, 1.149)	0.1150		
Intrapartum blood loss per 100 ml	0.789 (0.646, 0.962)	0.0191	0.756 (0.614, 0.930)	0.0081
Occurrence of fetal anomalies (Ref: No)	0.179 (0.039, 0.820)	0.0267	0.148 (0.030, 0.722)	0.0182
Use of support services on breastfeeding				
Help from lactation consultant during hospital stay (Ref: No)	1.772 (0.977, 3.214)	0.0596		
Help from ward nurse during hospital stay (Ref: No)	1.304 (0.666, 2.553)	0.4382		
Attended lactation clinic (Ref: No)	3.105 (0.397, 24.306)	0.2804		
Contacted hospital helpline (Ref: No)	0.620 (0.118, 3.266)	0.5725		
Did you receive any of these support services on breastfeeding or giving breast milk to your baby? (Ref: No)	3.160 (1.217, 8.207)	0.0181	3.304 (1.210, 9.020)	0.0197
Satisfaction with birth experience (Ref: Satisfied)		0.3410+		
Extremely satisfied	0.970 (0.449, 2.092)	0.5452		
Very satisfied	1.106 (0.575, 2.127)	0.2763		
Unsatisfied	1.608 (0.448, 5.773)	0.1800		
Very unsatisfied	0.169 (0.027, 1.057)	0.0406		

## Discussion

In our study, we have found that ethnic differences are independently associated with the likelihood to breastfeed for at least 5 to 9 weeks postpartum. Participants with no fetal anomalies, lesser intrapartum blood loss, lower state anxiety score and who received support or guidance on breastfeeding are more likely to be breastfeeding at 5 to 9 weeks postpartum. We did not find any significant associations between EPDS scores and the likelihood of breastfeeding at 5 to 9 weeks postpartum.

The 2011 National Breastfeeding Survey for Singapore showed that the prevalence of breastfeeding differed across the various ethnic groups, with higher prevalence found in the Chinese and Indians as compared to Malays and other ethnicities [1]. Similarly, Pang et al. conducted a cohort study involving 1030 Singaporean women in early pregnancy and found that the prevalence of breastfeeding at 6 months postpartum varies among the different ethnic groups even after adjusting for maternal education [16]. In our study, we found similar prevalence (79.1% vs 79.9%) of breastfeeding at 5 to 9 weeks postpartum as compared to the National Breastfeeding Survey in 2011 [1]. The ethnic differences and effects on breastfeeding at 5 to 9 weeks postpartum in our study also mirror those found in the National Breastfeeding Survey and Pang et al.’s study. Some barriers to breastfeeding which might account for such ethnic differences include the lack of social and cultural acceptance and support, language barriers, and lifestyle choices [17].

The Baby Friendly Hospital Initiative was jointly launched by the World Health Organization and the United Nations Children’s Fund in 1991 as a global effort to implement practices that promote, protect and support breastfeeding [18]. This includes improved support of breastfeeding in hospitals, actions to protect breastfeeding by national policy implementation, and public promotion campaigns. KKH was accredited as a baby-friendly hospital under the Initiative from 2014. We have also developed multiple support services to encourage breastfeeding, including training for obstetric ward nurses, arranging a visit to the breastfeeding dyad from a lactation consultant while in hospital and establishing a pre- and postnatal lactation clinic. Our participants also had access to a lactation telephone helpline and to the lactation consultant via phone after discharge. Other support services include the nationwide Breastfeeding Mothers’ Support Group Helpline and the Joyful Parenting and Breastfeeding Helpline. We found that participants who received support services on breastfeeding were 3.3 times more likely to be breastfeeding at 5 to 9 weeks postpartum compared to those who did not. This is however confounded by the fact that those participants who are more likely to want to breastfeed also tend to seek out such support services. Nonetheless, published literature have supported the efficacy of support services on the inclination to breastfeed and its continued duration postpartum [19, 20].

Our study showed an inverse association between STAI scores at baseline and breastfeeding at 5 to 9 weeks postpartum. Participants with more postpartum anxiety were more likely to stop breastfeeding by 5 to 9 weeks. In a systematic review by Fallon et al. on postpartum anxiety and infant-feeding outcomes, the authors found that women with postpartum anxiety are less likely to breastfeed exclusively and more likely to stop breastfeeding earlier [2]. In those who do breastfeed, postpartum anxiety reduces self-efficacy, increases breastfeeding difficulties, and may negatively affect breastfeeding behaviors and even breast milk composition. Heterogeneous outcomes and methodological limitations however limit the ability to compare across the studies in the review. Our findings are consistent with those found in similar studies such as a study in Pennsylvania which showed that the positive STAI score of 192 parturients were associated with poorer breastfeeding outcomes in the first 6 months postpartum [21].

We found that intrapartum blood loss is negatively associated with breastfeeding at 5 to 9 weeks postpartum. We hypothesize that the amount of blood lost intrapartum is an indicator of the difficulty and complexity of the delivery process. To the participants, this can translate into birth traumas that can become a source of psychological distress. It has been shown in other studies that traumatic stressors can influence and truncate the duration of breastfeeding in mothers [22, 23]. Additionally, it can be argued that substantial hemorrhages can lead to delayed initiation of breastfeeding. This could be due to the mothers feeling fatigued from anemia, or that the mothers had to receive specialized care postpartum in a high dependency unit or in an intensive care unit. A delayed initiation of breastfeeding has been shown to increase the risk of breastfeeding cessation. Thompson et al. conducted a study of 206 mothers looking at associations between postpartum hemorrhage and breastfeeding experiences, and showed that women with greater blood loss are more likely to have delayed initiation of breastfeeding and shortened duration of breastfeeding [24]. Similarly, Brown and Jordan found, in their cross-sectional survey of 284 mothers, that postpartum hemorrhage was significantly associated with shorter breastfeeding duration [25].

The presence of fetal anomalies having a negative impact on breastfeeding at 5 to 9 weeks postpartum could be explained by the increased psychosocial burden placed on the families. Mothers of infants with fetal anomalies may find it more difficult to feed their babies and have increased distress over the overall care of their newborns [26–28]. This burden could be intensified if the newborns had been admitted into the intensive care unit [29]. That being said, it is still possible to improve breastfeeding outcomes in newborns with congenital anomalies so long as the primary care team adopts the necessary measures [30]. Torowicz et al.’s prospective cohort study of 62 mothers has shown that the attitudes of the institution and the advocacy for breastfeeding are key for the initiation and maintenance of breastfeeding in mothers who have infants with complex congenital heart diseases [31].

Our study did not show any significant associations between EPDS scores and breastfeeding at 5 to 9 weeks postpartum. This finding differs from those seen in other studies, which show that higher EPDS scores were associated with cessation of breastfeeding [32–34]. Likewise, a systematic review of existing literature on breastfeeding and maternal depression has shown that both pregnancy and postpartum depression predict shorter breastfeeding durations [35]. We hypothesize that the lack of an association in our study could be the short follow-up period (5 to 9 weeks postpartum) as opposed to the time point assessments adopted in other studies.

This study is limited by confounders present in our study such as participants with preconceived intentions to breastfeed and participants with background prenatal anxiety and depression. Other considerations such as smoking status and body mass index that may serve as potential confounders were also not taken into account in our study. Another limitation is our broader inclusion criteria of participants with 59 who breastfed exclusively and 97 who breastfed non-exclusively, rather than specifically analysing participants who breastfed exclusively. Furthermore, we only looked at participants who have received labour epidural analgesia. In addition, our study conducted follow ups at 5 to 9 weeks postpartum, and did not follow up at 6 months postpartum. While breastfeeding is recommended for at least the first 6 months, we recognise that psychological vulnerabilities such as anxiety and depression are prevalent in the weeks following labour, thus our selection of follow up time points. We only analysed participants who completed all stages of this study, and we did not analyse the differences between these participants and those with incomplete stages. Finally, we found that insufficient milk supply was a common reason for cessation of breastfeeding according to responses. However, we did not explore the milk insufficiency in these women.

## Conclusion

Breastfeeding is substantially beneficial to both mother and child. Given the importance of breastfeeding, it is essential to identify risk factors that can influence breastfeeding outcomes. In our study, we have ascertained several determinants that have significant impact on breastfeeding at 5 to 9 weeks postpartum. Healthcare providers could utilize this risk stratification to identify parturients likely to have poorer breastfeeding outcomes and undertake interventions that may help safeguard optimization of breastfeeding outcomes and parturient care.

## Additional files

Additional file 1: Breastfeeding Questionnaire. This is a copy of the Breastfeeding Questionnaire which was conducted via a telephone interview to the participant at 5 to 9 weeks postpartum. (PDF 42 kb)

Additional file 2: Receiver Operating Characteristic (ROC) curve. The Receiver Operating Characteristic (ROC) curve for the independent covariates for breastfeeding 5 to 9 weeks postpartum. The area under the curve is 0.7065. (TIFF 655 kb)

## Abbreviations

AUC: Area under the ROC curve
CI: Confidence interval
CIRB: Centralised Institutional Review Board
COLEUS: Collaborative Outcomes with Labour Epidural Use Study
EPDS: Edinburgh postnatal depression scale
KKH: KK Women’s and Children’s Hospital
OR: Odds ratio
PCS: Pain catastrophizing scale
PSS: Perceived stress scale
ROC: Receiver operating characteristic
SAS: Statistical analysis software
SD: Standard deviation
SFMPQ: Short form McGill pain questionnaire
STAI: State-Trait anxiety inventory

## References

[1] Chua L, Win AM. Prevalence of breastfeeding in Singapore. In: Statistics Singapore Newsletter. 2013. https://www.singstat.gov.sg/-/media/files/publications/society/ssnsep13-pg10-14.pdf. Accessed 30 Aug 2018.

[2] Fallon V, Groves R, Halford JC, Bennett KM, Harrold JA (2016). Postpartum anxiety and infant-feeding outcomes: a systematic review. J Hum Lact.

[3] Dennis CL, McQueen K (2009). The relationship between infant-feeding outcomes and postpartum depression: a qualitative systematic review. Pediatrics.

[4] Henderson JJ, Evans SF, Straton JA, Priest SR, Hagan R (2003). Impact of postnatal depression on breastfeeding duration. Birth.

[5] Gunning MD, Denison FC, Stockley CJ, Ho SP, Sandhu HK, Reynolds RM (2010). Assessing maternal anxiety in pregnancy with the state-trait anxiety inventory (STAI): issues of validity, location and participation. J Reprod Infant Psychol.

[6] Grafton KV, Foster NE, Wright CC (2005). Test-retest reliability of the short-form McGill pain questionnaire: assessment of intraclass correlation coefficients and limits of agreement in patients with osteoarthritis. Clin J Pain.

[7] Gibson J, McKenzie-McHarg K, Shakespeare J, Price J, Gray R (2009). A systematic review of studies validating the Edinburgh postnatal depression scale in antepartum and postpartum women. Acta Psychiatr Scand.

[8] Lee EH (2012). Review of the psychometric evidence of the perceived stress scale. Asian Nurs Res.

[9] Osman A, Barrios FX, Gutierrez PM, Kopper BA, Merrifield T, Grittmann L (2000). The pain catastrophizing scale: further psychometric evaluation with adult samples. J Behav Med.

[10] Chen H, Bautista D, Ch'ng YC, Li W, Chan E, Rush AJ (2013). Screening for postnatal depression in Chinese-speaking women using the Hong Kong translated version of the Edinburgh postnatal depression scale. Asia Pac Psychiatry.

[11] Wilson EB (1927). Probable inference, the law of succession, and statistical inference. J Am Stat Assoc.

[12] Brown LD, Cai TT, DasGupta A (2001). Interval estimation for a binomial proportion. Stat Sci.

[13] Peduzzi P, Concato J, Feinstein AR, Holford TR (1995). Importance of events per independent variable in proportional hazards regression analysis II. Accuracy and precision of regression estimates. J Clin Epidemiol.

[14] Concato J, Peduzzi P, Holford TR, Feinstein AR (1995). Importance of events per independent variable in proportional hazards analysis I. Background, goals, and general strategy. J Clin Epidemiol.

[15] Vittinghoff E, McCulloch CE (2007). Relaxing the rule of ten events per variable in logistic and cox regression. Am J Epidemiol.

[16] Pang WW, Aris IM, Fok D, Soh SE, Chua MC, Lim SB (2016). Determinants of breastfeeding practices and success in a multi-ethnic asian population. Birth.

[17] Jones KM, Power ML, Queenan JT, Schulkin J (2015). Racial and ethnic disparities in breastfeeding. Breastfeed Med.

[18] World Health Organization, UNICEF (2009). Baby-friendly Hospital Initiative: Revised, Updated and Expanded for Integrated Care.

[19] Renfrew MJ, McCormick FM, Wade A, Quinn B, Dowswell T (2012). Support for healthy breastfeeding mothers with healthy term babies. Cochrane Database Syst Rev.

[20] Taveras EM, Capra AM, Braveman PA, Jensvold NG, Escobar GJ, Lieu TA (2003). Clinician support and psychosocial risk factors associated with breastfeeding discontinuation. Pediatrics.

[21] Paul IM, Downs DS, Schaefer EW, Beiler JS, Weisman CS (2013). Postpartum anxiety and maternal-infant health outcomes. Pediatrics.

[22] Thompson RE, Kildea SV, Barclay LM, Kruske S (2011). An account of significant events influencing Australian breastfeeding practice over the last 40 years. Women Birth.

[23] Beck CT, Watson S (2008). Impact of birth trauma on breast-feeding: a tale of two pathways. Nurs Res.

[24] Thompson JF, Heal LJ, Roberts CL, Ellwood DA (2010). Women's breastfeeding experiences following a significant primary postpartum haemorrhage: a multicentre cohort study. Int Breastfeed J.

[25] Brown A, Jordan S (2013). Impact of birth complications on breastfeeding duration: an internet survey. J Adv Nurs.

[26] Medoff-Cooper B, Naim M, Torowicz D, Mott A (2010). Feeding, growth, and nutrition in children with congenitally malformed hearts. Cardiol Young.

[27] Rempel GR, Ravindran V, Rogers LG, Magill-Evans J (2013). Parenting under pressure: a grounded theory of parenting young children with life-threatening congenital heart disease. J Adv Nurs.

[28] Svavarsdottir EK, McCubbin M (1996). Parenthood transition for parents of an infant diagnosed with a congenital heart condition. J Pediatr Nurs.

[29] Pinelli J (2000). Effects of family coping and resources on family adjustment and parental stress in the acute phase of the NICU experience. Neonatal Netw.

[30] Spatz DL (2005). Report of a staff program to promote and support breastfeeding in the care of vulnerable infants at a children’s hospital. J Perinat Educ.

[31] Torowicz DL, Seelhorst A, Froh EB, Spatz DL (2015). Human milk and breastfeeding outcomes in infants with congenital heart disease. Breastfeed Med.

[32] Akman İ, Kuscu MK, Yurdakul Z, Özdemir N, Solakoğlu M, Orhon L (2008). Breastfeeding duration and postpartum psychological adjustment: role of maternal attachment styles. J Paediatr Child Health.

[33] Dennis CL, McQueen K (2007). Does maternal postpartum depressive symptomatology influence infant feeding outcomes?. Acta Paediatr.

[34] Nishioka E, Haruna M, Ota E, Matsuzaki M, Murayama R, Yoshimura K (2011). A prospective study of the relationship between breastfeeding and postpartum depressive symptoms appearing at 1–5months after delivery. J Affect Disord.

[35] Dias CC, Figueiredo B (2015). Breastfeeding and depression: a systematic review of the literature. J Affect Disord.

