# Supplementary material for: Investigating factors associated with success of breastfeeding in first-time mothers undergoing epidural analgesia: a prospective cohort study
Source: Int Breastfeed J. 2018 Sep 5;13:42. doi: 10.1186/s13006-018-0184-7 (PMC6125871; doi:10.1186/s13006-018-0184-7)
Supplement: Supplementary file 1 — Breastfeeding Questionnaire. This is a copy of the Breastfeeding Questionnaire which was conducted via a telephone interview to the participant at 5 to 9 weeks postpartum. (PDF 42 kb) [file 13006_2018_184_MOESM1_ESM.pdf]

12. Breastfeeding

Study ID __________________________________

Is data collection completed? ◯ Yes ◯ No

If No, please specify the reason:
◯ Uncontactable
◯ Declined Survey
◯ Blank
◯ Others

If Others, please specify the reason: __________________________________

Telephone follow-up at 5-9 weeks Postpartum

Date of Phone call: __________________________________
((DD/MM/YYYY))

Phone call by:
◯ Kirsten Sim
◯ Ng Jing Wen
◯ Denise Anne
◯ Wong Anng Anng
◯ Yvonne Yong

1. Did you receive any of these support services on breastfeeding or giving breast milk to your baby? (may select more than1)

☐ Not at all
☐ Help from ward nurses during my stay in hospital
☐ Help from Lactation Consultant during my stay in hospital
☐ Received a phone call from postnatal ward after discharge
☐ Contacted the KKH Ask-a-Nurse Helpline
☐ Contacted the KKH Lactation Consultant
☐ Attended KKH Lactation Clinic
☐ Attended KKH 24hrs O&G Clinic
☐ Breastfeeding Mothers' Support Group Helpline
☐ Joyful Parenting & Breastfeeding Helpline
☐ Others

1.If others, pls specify: __________________________________

2. Are you still breastfeeding or giving breast milk your baby?

◯ Yes (go to question 3 and 4) ◯ No (go to question 6 and 7)

3. How was breast milk fed to your baby in the last 24 hours? (may select more than 1)

☐ Direct breastfeeding
☐ Expressed breast milk through bottle
☐ Expressed breast milk through syringe/cup/spoon

4. What were you feeding your baby in the last 24 hours? (may select more than one answer):

☐ Breast milk ☐ Infant formula (Answer Q5) ☐ Water ☐ Solid food

5. If you are breastfeeding or giving breast milk partially to your baby, when you started with infant formula: __________________________________
(DD-MM-YYYY)

Age in weeks: __________________________________
(weeks (note: Round up))

Reasons for introducing infant formula (may select more than 1 answer):

- ☐ Fatigue/Tiredness
- ☐ Sore nipples
- ☐ Painful breast
- ☐ Insufficient milk supply
- ☐ Feeding difficulty (latching problem)
- ☐ Choose to partial breastfeed
- ☐ Returning to work after maternity leave
- ☐ No Breastfeeding facilities at workplace
- ☐ Encouraged by family members
- ☐ Encouraged by Confinement nanny
- ☐ Infant's Medical Reasons:
- ☐ Mother's Medical Reasons:
- ☐ Others, please specify

Pls specify, Infant's Medical Reasons: ______________________________

Pls specify, Mother's Medical Reasons: ______________________________

Others, please specify ______________________________

6. If you have stopped either breastfeeding or giving breast milk to your baby when did you stop breastfeeding? ______________________________ ((DD/MM/YYYY))

7. Reasons for stopping breastfeeding (may select more than 1 answer):

- ☐ Fatigue/ Tiredness
- ☐ Sore nipples
- ☐ Painful breast
- ☐ Insufficient milk supply
- ☐ Feeding difficulty (latching problem)
- ☐ Choose to stop breastfeeding
- ☐ Return to work after maternity leave
- ☐ No Breastfeeding facilities at workplace
- ☐ Encouraged by family members
- ☐ Encouraged by confinement nanny
- ☐ Infant's Medical Reasons:
- ☐ Mother's Medical Reasons:
- ☐ Others:

Pls specify Infant's Medical Reasons: ______________________________

Pls specify Mother's Medical Reasons: ______________________________

Specify Others: ______________________________
