# Supplementary figures and images for: Investigating factors associated with success of breastfeeding in first-time mothers undergoing epidural analgesia: a prospective cohort study
Source: Int Breastfeed J. 2018 Sep 5;13:42. doi: 10.1186/s13006-018-0184-7 (PMC6125871; doi:10.1186/s13006-018-0184-7)

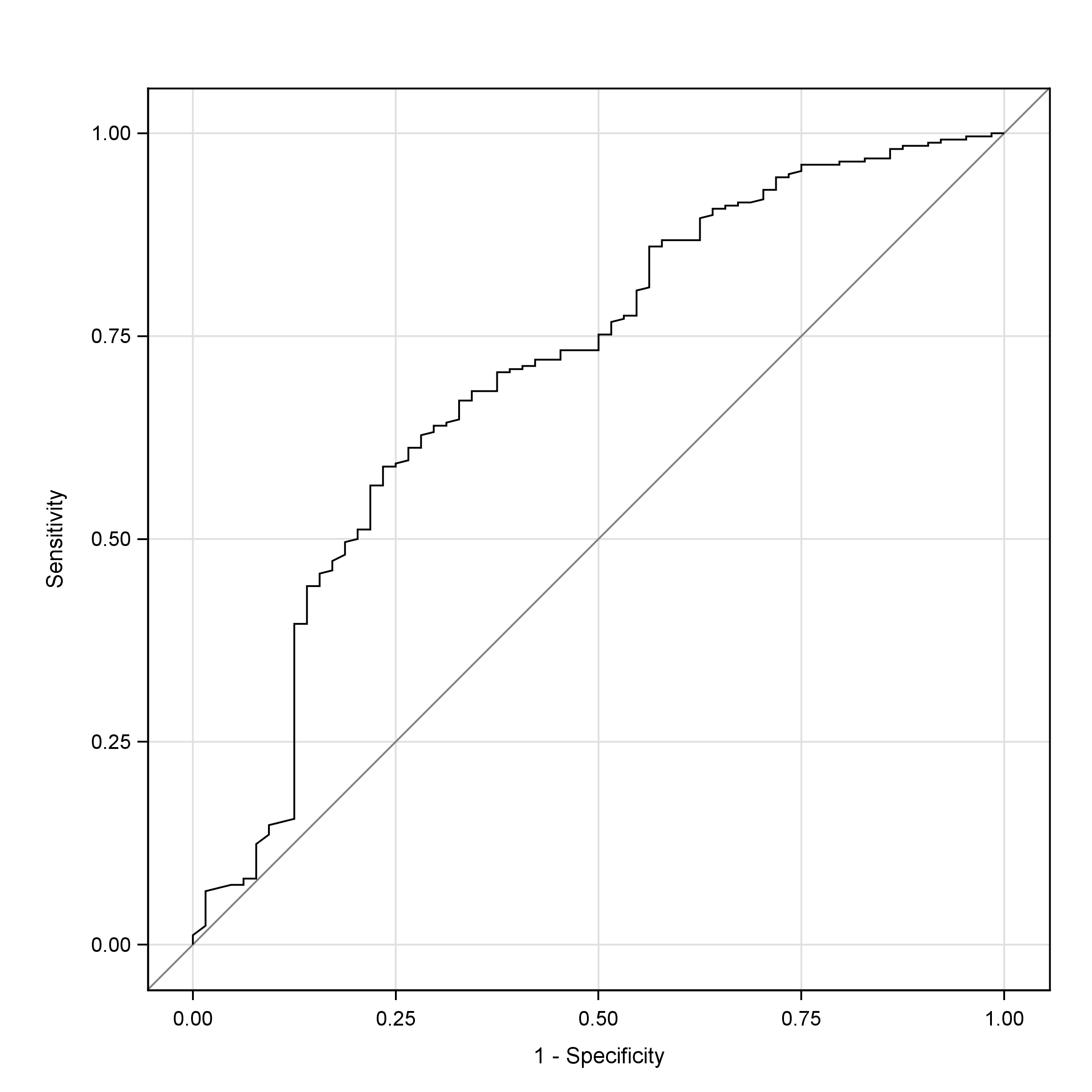

Supplement: Supplementary file 2 — Receiver Operating Characteristic (ROC) curve. The Receiver Operating Characteristic (ROC) curve for the independent covariates for breastfeeding 5 to 9 weeks postpartum. The area under the curve is 0.7065. (TIFF 655 kb) [file 13006_2018_184_MOESM2_ESM.tiff]
